# Highlights of the Latest Developments in Radiopharmaceuticals for Infection Imaging and Future Perspectives

**DOI:** 10.3389/fmed.2022.819702

**Published:** 2022-02-11

**Authors:** Ekaterina Dadachova, Drauzio E. N. Rangel

**Affiliations:** ^1^College of Pharmacy and Nutrition, University of Saskatchewan, Saskatoon, SK, Canada; ^2^Universidade Brazil, São Paulo, Brazil

**Keywords:** infection imaging, antibiotics, anti-fungals, antimicrobial peptides, antibodies, HIV, PET, SPECT

## Abstract

COVID-19 pandemic has heightened the interest toward diagnosis and treatment of infectious diseases. Nuclear medicine with its powerful scintigraphic, single photon emission computer tomography (SPECT) and positron emission tomography (PET) imaging modalities has always played an important role in diagnosis of infections and distinguishing them from the sterile inflammation. In addition to the clinically available radiopharmaceuticals there has been a decades-long effort to develop more specific imaging agents with some examples being radiolabeled antibiotics and antimicrobial peptides for bacterial imaging, radiolabeled anti-fungals for fungal infections imaging, radiolabeled pathogen-specific antibodies and molecular engineered constructs. In this opinion piece, we would like to discuss some examples of the work published in the last decade on developing nuclear imaging agents for bacterial, fungal, and viral infections in order to generate more interest among nuclear medicine community toward conducting clinical trials of these novel probes, as well as toward developing novel radiotracers for imaging infections.

## Introduction

COVID-19 pandemic has heightened the interest toward diagnosis and treatment of infectious diseases. Nuclear medicine with its powerful scintigraphic, single photon emission computer tomography (SPECT) and positron emission tomography (PET) imaging modalities has always played an important role in diagnosis of infections and distinguishing them from the sterile inflammation. The classical tools developed decades ago but still widely used in the clinic today are ^111^In-labeled leukocyte imaging for most indications, ^67^Ga for imaging of opportunistic infections, pulmonary inflammation and interstitial nephritis and 2-Deoxy-2-[^18^F]fluoroglucose ([^18^F]FDG) for spinal osteomyelitis, vasculitis, sarcoidosis, and fever of unknown origin (1, 2) as well as for identifying cardiovascular device infection (3).

In addition to these clinically available radiopharmaceuticals there has been a decades-long effort to develop more specific imaging agents with some examples being radiolabeled antibiotics and antimicrobial peptides for bacterial imaging, radiolabeled anti-fungals for fungal infections imaging, radiolabeled pathogen-specific antibodies and molecular engineered constructs. Several excellent reviews on these agents which are primarily still in the preclinical stage have appeared in the last decade (4–6). In this opinion piece, we would like to discuss some examples of the work published in the last decade on developing nuclear imaging agents for bacterial, fungal and viral infections in order to generate more interest among nuclear medicine community toward conducting clinical trials of these novel probes, as well as toward developing novel radiotracers for imaging infections.

## Imaging Bacterial Infections With Radiolabeled Antibiotics, Sugars, Antibacterial Peptides and Bacterial Antigen-Specific Antibodies

Imaging of bacterial infections is by far the most developed area of infection imaging. Multiple antibiotics with a variety of mechanisms of action such as ciproflaxicin (7, 8) and nitrofuryl thiosemicarbazone (7), fluoroquinolone (9), isoniazid (10), ofloxacin (11, 12), cephalosporin (13), clindamycin (14), doxycycline (15), ceftizoxime (16, 17), cefotaxime (18), tinidazole (19), sulfadiazine (20), tazobactam (21) and metronidazole (22) were radiolabeled with ^99m^Tc to enable SPECT imaging in the pre-clinical models of infection. The majority of the authors employed Sn(+2) salts as the reducing agents for ^99m^Tc which produced [^99m^Tc-O](3+) core, while in several publications ^99m^Tc carbonyl or [^99m^Tc-N](2+) cores were utilized (7, 8, 11). The smaller size of ^99m^Tc carbonyl core might be less perturbing to the molecular structure of antibiotic molecules. Satpati et al. used a smaller sized radiometal, PET enabling ^68^Ga, for labeling of ciprofloxacin via two common bifunctional chelating agents 2,2′,2″,2^‴^-(1,4,7,10-Tetraazacyclododecane-1,4,7,10-tetrayl)tetraacetic acid (DOTA) and 2,2′,2″-(1,4,7-triazacyclononane-1,4,7-triyl)triacetic acid (NOTA) (23). However, both of these chelating agents are quite bulky macrocycles. In this regard, Northrup et al. (24) considered labeling antibiotics with radiometals problematic as it can affect the probe entering the bacterium, or interfere with the binding to the intracellular target.

To avoid radiometals, one can potentially use PET-enabling “organic” radionuclides such as ^13^N or ^11^C, or ^18^F as proposed by Naqvi and Drlica (25) which should not perturb the molecular structure of an antibiotic due to the latter small atomic radius. Wang et al. labeled PT119, a potent *Staphylococcus aureus* enoyl-ACP reductase (saFabI) inhibitor, with ^11^C and performed PET imaging of *S. aureus-*infected mice with this agent (26). Mota et al. performed PET imaging of ^18^F-linezolid in a mouse model of pulmonary tuberculosis (TB) (27). Liu et al. took the use of “organic” radionuclides one step further by radiolabeling anti-TB chemotherapeutics isoniazid (INH), rifampicin (RIF), and pyrazinamide (PZA) with ^11^C and performing the whole body PET imaging of each radiolabeled drug in baboons (28). They also used the polyethylene glycol (PEG)ylated forms of the same anti-TB drugs for PET imaging and made a conclusion about the potential utility of the PEGylated isoniazid conjugates as long-circulating carriers for improved therapy of TB (28). Of course, when choosing a radionuclide for antibiotics labeling, researchers have to take into consideration the radionuclide cost, its availability and accessibility of the SPECT or PET imaging equipment in their institutions.

The majority of the infection models used in the pre-clinical studies of radiolabeled antibiotics were murine, but some groups used rabbits (10, 14, 19, 20), as the immune system of rabbits is considered to be closer to a human immune system (29). *Staphylococcus aureus* was used most often to induce infection in experimental animals, but other bacteria such as *Mycobaterium tuberculosis* (10), *Bacteroides fragilis* and *Dentamoeba fragilis* (19), *E. coli* (20, 22), *Pseudomonas aeruginosa* and *Salmonella enterica* (21) which are also important from the clinical perspective were utilized as well. Many groups not only imaged the localization of the radiolabeled antibiotics with SPECT or PET but also quantified the images and in some case performed the biodistribution in addition to imaging. Kakkar et al. reported retention of ^99m^Tc-labeled isoniazid at the sites of *M. tuberculosis* infection in rabbits for 72 h (10).

In *S. aureus* mouse model the biodistribution results showed that accumulation of ^99m^Tc-ofloxacin in the infected muscle reached target to non-target (T/NT) ratio of 2 at 4 h post injection (11) while ^68^Ga-ciprofloxacin tested in rats infected with *S. aureus* demonstrated T/NT ratio of 3–6 at 2 h depending on the chelating agent used (23). In *E. coli* rabbit model the accumulation of ^99m^Tc-metronidazole at 1 h post injection reached T/NT ratio of 5.57 (22). Interestingly, the authors of all reviewed here manuscripts concluded that their results were encouraging. It is not clear at the moment what stands in a way of clinical translation of these relatively cheap radiopharmaceuticals. It might be the single digit values for absolute uptake in the infected sites which is lower than usually reported numbers for tumor uptake in the preclinical studies. However, in human patients the situation might be different and first in man clinical trials of radiolabeled antibiotics seem to be warranted.

Antimicrobial peptides (AMPs), described as natural microbicides, accumulate at the sites of the bacterial infection due to their positive charge which is electrostatically attracted to the negative charged surface of the bacterial cells. This feature makes them attractive as potential infection imaging agents (30). Among multiple AMPs ubiquicidin (UBI) seems to be one of the most widely used in the preclinical work and in clinical trials. It has been labeled with ^99m^Tc via hydrazinonicotinamide (HYNIC) or other chelating agents to enable SPECT imaging (31, 32) but relatively recent availability of PET-enabling ^68^Ga has made the labeling with the latter supplanting other radionuclides due to the good match between short physical half-life of ^68^Ga (68 min) and short plasma half-life of ubiquicidin and other AMPs (33, 34). Other recently described AMPs-radionuclide combinations include [^99m^Tc-HYNIC/EDDA]-MccJ25 (35); ^99m^Tc-human beta-defensin 3 (HBD-3) (36); ^64^Cu-labeled cationic peptides HLys-DOTA, synthesized as the D-isomer and AB1-HLys-DOTA, synthesized with an unnatural aminoacid to slow down the blood clearance (37); and ^68^Ga-DOTA-CF-17 (38).

The advantage of AMPs over radiolabeled antibiotics is that AMP are larger molecules which should allow attachment of radiolabels without loss of the ability to bind bacteria in a specific manner; the disadvantage is their fast blood clearance due to enzymatic destruction and also high kidney uptake. Evaluation of AMPs in murine models of *S. aureus* (34, 36–38), mice with *E. coli* infection (35), *P. aeruginosa* (38) demonstrated the highest target to non-target ratios of 2–6 reached from 45 to 120 min post administration of radiolabeled peptides, showing their ability to distinguish the infected tissues from sterile inflammation. Auletta et al. performed side by side comparison of ^99m^Tc-Ubiquicidin 29-41, ^99m^Tc-ciprofloxacin and ^99m^Tc-ciprofloxacin dithiocarbamate (CS2) *in vitro* and in mice with *S. aureus* and *E.coli* infections (32). They concluded that ^99m^Tc-UBI and ^99m^TcN-Cipro CS2 showed good *in vivo* binding to *E. coli* only while ^99m^Tc-Ciprofloxacin demonstrated good *in vivo* binding to both *E. coli* and *S. aureus*.

The radiolabeled antimicrobial peptides have been tested in first in man type of clinical trials. Kamaleshwaran reported the diagnosis of knee prosthesis infection with ^99m^Tc-ubiquicidin scintigraphy and its comparison with FDG PET/CT (31). Both of the traces were able to diagnose the infection. Ebenhan et al. evaluated ^68^Ga-NOTA-UBI with PET/CT in two healthy volunteers and 3 patients with suspected bacterial infection (32). ^68^Ga-NOTA-UBI was able to diagnose bone- and soft-tissue infection in all three patients. Radiation dosimetry performed as part of this trial identified bladder wall as a dose-limiting tissue (185 μSv/MBq), followed by the kidneys (23 μSv/MBq). The total absorbed body dose was below 7 μSv/MBq with the effective dose being ~17 μSv/MBq. The authors concluded that ^68^Ga-NOTA-UBI is a promising diagnostic technique for infection imaging.

The problem of distinguishing between a sterile inflammation and a bacterial infection is important, especially in post-operative patients. It prompted the studies of imaging molecules which would target bacteria-specific metabolic pathways. Recent studies from several groups have demonstrated that it is possible to radiolabel with ^18^F sugars and sugar alcohols that are not efficiently metabolized by humans, such as 2-deoxy-2-[^18^F]fluorosorbitol (^18^F-FDS), 6-[^18^F]fluoromaltose, and 6″-[^18^F]fluoromaltotriose without interfering with their uptake by bacteria thus creating the probes for PET imaging (39–41). The approach of radiolabeling sugars in the SPECT arena has also been pursued—Shukla et al. radiolabeled hydroxypropyl-β-cyclodextrin (HPβCD), a oligosaccharide derivative, with ^99m^Tc to assist with the differentiation between loosening of prosthesis due to septic or aseptic course in patients (42). Nanoparticles ^99m^Tc HPβCD in the quantity of 0.5–1.0 mg were injected in human subjects with clinically confirmed infected knee joints, with clear distinction between the septic and aseptic loosening being observed on SPECT images (42), thus providing first in man clinical experience with radiolabeled sugars for bacterial infection imaging.

Some interesting insights into imaging of bacterial infections with the radiolabeled antibodies have been reported recently. Pickett et al. evaluated a human monoclonal antibody (mAb) which binds to the Gram-positive bacterial surface molecule lipoteichoic acid (LTA) by radiolabeling this anti-LTA mAb SAC55 with ^89^Zr and investigating its utility as a PET agent for diagnosis of infection in a mouse model of prosthetic joint infection (PJI) (43). Twenty four hours post-injection ^89^Zr-SAC55 displayed significantly higher uptake at *S. aureus*-infected prosthesis sites than at the sterile prosthesis sites. Importantly, ^89^Zr-SAC55 uptake at the infected site was also higher than that of the control non-specific antibody, thus demonstrating specificity and selectivity. Foss et al. pursued the imaging of *M. tuberculosis* bacteria whose mostly intracellular location makes it difficult to detect it with the antibodies (44). The strategy used by the authors was to develop an antibody (mAb 3d29) which recognizes the tissue-bound terminal processing fragments iC3b and C3d of C3 complement but not the native circulating C3 or tissue-bound C3b. SPECT with ^125^I-3d29 mAb showed lesions in the lungs and spleens of the aerosol-infected with *M. tuberculosis* C3HeB/FeJ mice at 24 and 48 h post-administration. These lesions colocalized with granulomas detected by CT, and the antibody was detected in the cytoplasm of macrophages, and within alveolar epithelial cells which is consistent with the location of internalized *M. tuberculosis*. Very insignificant uptake of the ^125^I-3d29 mAb in the lungs and spleen of the healthy mice and 3.5:1 ratio of increased uptake of ^125^I-3d29 mAb over isotype control mAb in infected lungs allowed the authors to conclude that ^125^I-3d29 can be utilized for localization of *M. tuberculosis* infection 24 h after administration.

## Imaging of Fungal Infections With Radiolabeled Anti-Fungal Drugs and Antibodies

Invasive fungal infections especially in immunocompromised patients are on the rise worldwide and there is a need to diagnose them in an expedited manner as the growth of fungal cultures obtained from patients might take weeks which is often not feasible for critically ill patients (45). Page et al. aimed at developing cost-efficient and broadly useful tracers for pulmonary mold infections by radiolabeling amphotericin B (AMB) with ^99m^Tc and ^68^Ga. The radiolabeled antifungal was stable in human serum and both tracers accumulated specifically in Transwell inserts infected with *Aspergillus fumigatus, Rhizopus arrhizus*, and other clinically relevant mold pathogens in comparison with uninfected inserts or inserts infected with bacterial controls (46). This encouraging *in vitro* study warrants further *in vivo* evaluation of radiolabeled amphotericin B for molecular imaging of invasive mycoses.

Fungi capable of human pathogenesis have developed special siderophore metabolism as a way to pirate the iron in the challenging iron-free environment of a host. Siderophore metabolism of *A. fumigatus* was investigated by Petrik et al. and Haas et al. by making N^2^-acetylated derivative triacetylfusarinine C (^68^Ga-TAFC) and ^68^Ga-ferrioxamine E (^68^Ga-FOXE) PET-enabling molecules (47, 48). These probes showed fast uptake in the lungs of A. fumigatus-infected rats, low accumulation in sterile inflammation and no uptake in bacterial abscess (47, 48). The drawback of this approach is the absence of siderophore metabolism in several clinically relevant fungi, which will require to first diagnose the fungal infection by a different technique.

Other groups pursued the imaging approach based on fungal antigen-specific antibodies. Davies et al. developed a humanized mAb hJF5 binds to the antigenic determinant β1,5-galactofuranose (Gal*f*) present in a diagnostic mannoprotein antigen released by the pathogen during invasive growth in the lung while this epitope Gal*f* is absent in mammalian carbohydrates (49). The authors performed PET imaging of *A. fumigatus* lung infection in mice with [^64^Cu]NODAGA-hJF5 mAb. *In vivo* quantification demonstrated higher uptake of [^64^Cu]NODAGA-hJF5 mAb in the lungs of infected mice in comparison with the PBS-treated controls or with infected and control animals administered with free radionuclude [^64^Cu]Cl_2_. In this regard, uptake of [^64^Cu]NODAGA-hJF5 in the lungs of infected mice 48 h post administration was 17.1 ± 2.5%ID/cc while that in PBS-treated control mice was 9.4 ± 1.1%ID/cc. Henneberg et al. continued development of hJF5 as an imaging agent not only for detection of *A. fumigatus* infections but also for monitoring the response to azole treatment (50). For this purpose they dual labeled hJF5 with ^64^Cu and fluorophore and performed immunoPET/MRI *in vivo* in a neutropenic mouse model of *A. fumigatus* infection. They concluded that antibody-guided approach revealed that early drug intervention is critical to prevent complete invasion of the lungs by the pathogen, and showed the potential of immunoPET for diagnosis and monitoring *A. fumigatus* infections.

## Imaging of Viral Infections

Viral infections with human immunodeficiency virus (HIV), influenza A and SARS-CoV-2 result in deadly acute disease such as AIDS, influenza and COVID-19, respectively, while some viral infections such as human papilloma virus HPV-16 and HPV-18 viruses are a causation behind several types of cancer, e.g., cervical, anal, and head and neck cancers. In this regard, nuclear imaging can be indispensable for non-invasive detection of virally infected cells, monitoring the treatment progression or side effects.

Immune system can recognize peptides on major histocompatibility molecules which helps to the eradicate infectious agents and even cancers. *In vivo* imaging of such responses could assist with evaluating the efficacy of immune interventions. and improve mechanistic understanding of immune responses. Woodham et al. utilized synTacs, which are dimeric major histocompatibility molecule scaffolds of defined composition, for imaging antigen-specific CD8(+) T cells *in vivo* (51). The authors imaged HPV16 E7-specific CD8 T cells with PAT by utilizing a ^89^Zr- or ^64^Cu-labeled HPV16 E7 peptide-loaded synTac in HPV16-positive tumors, following administration of a therapeutic vaccine. ImmunoPET with flu-specific synTac also produced positive results in imaging of influenza A virus (IAV) nucleoprotein-specific CD8 T cells in the lungs of IAV-infected mice. The authors have concluded that it is possible to visualize antigen-specific CD8+ T cell populations *in vivo*, which may play prognostic and diagnostic roles.

Some of the most pressing questions in HIV research—revealing viral dissemination in real-time and detecting HIV reservoirs during suppressive antiretroviral therapy (ART)—remain a challenge and are currently limited to blood sampling and biopsies. There is an enduring assumption in HIV field which, however, to the best of our knowledge is not based on any experimental data, that during ART virally infected cells do not express any HIV-specific antigens on their surface and thus, cannot be imaged *in vivo*. Song et al. genetically engineered simian immunodeficiency virus (SIV) to carry different imaging reporters with the purpose of demonstrating that *in vivo* imaging can be used to visualize SIV-transduced cells (52). Based on the expression of the reporter genes, the group was able to image and enumerate the SIV-transduced cells via vesicular stomatitis virus glycoprotein pseudotyping in a mouse model using bioluminescence imaging, PET-CT or MRI. In addition, they also engineered a chimeric EcoSIV for *in vivo* infection study and were able to prove that PET-CT can provide 3D images of the spatial location of as few as 10,000 SIV-infected cells. Santagelo et al. extended imaging of SIV reservoirs to non-human primate model (NHP) (53). The authors administrated that a radiolabeled with ^64^Cu pegylated SIV Gp120-specific mAb produced readily detectable signals during PET/CT imaging in the gastrointestinal and respiratory tract, lymphoid tissues and reproductive organs of viremic monkeys. These viral signals were reduced in aviremic NHPs but still detectable in colon, select lymph nodes, small bowel, nasal turbinates, the genital tract and lung, thus invalidating the assumption that virally infected cells cannot be imaged in patients on ART. Moreover, in elite controllers, virus was detected primarily in foci in the small bowel, select lymphoid areas and the male reproductive tract, as confirmed by quantitative reverse-transcription PCR (qRT-PCR) and immunohistochemistry. The authors concluded that immunoPET viral imaging has broad applications for the study of immunodeficiency virus pathogenesis, drug and vaccine development, and the potential for clinical translation. The presence of HIV gp41 protein on the surface of cells from HIV patients on different ART regimens was subsequently demonstrated *ex vivo* with radiolabeled mAbs to human HIV gp41 (54).

The urgency of imaging patients acutely ill with COVID-19 as well as following up post-COVID patients and those receiving vaccines, did not allow for time to create SARS-CoV-2 specific imaging agents. The domineering modality so far has been FDG-PET/CT covered in several excellent reviews (55, 56) while SPECT imaging also contributes, for example, toward imaging of pulmonary emboli in COVID patients (57). As COVID-19 will gradually become endemic, it is only a matter of time when COVID-specific PET and SPECT imaging agents are developed.

## Future Perspectives

### Problems Preventing Clinical Translation of Infection Imaging Agents

The field of infections imaging is still far behind oncology or neurology in investigating novel tracers in clinical trials (58). This is in spite of the rich armamentarium of novel tracers for imaging of bacterial, fungal and viral infections which preclinical research has already generated. One of the problems facing researchers who would like to translate their findings into the clinic, is the lack of the standardized infection models, which makes it difficult to compare the results generated by various research groups and to accumulate the preclinical data needed for regulatory submissions leading to a clinical trial. Many microbiologists and infectious disease physicians are unaware of the existence of the wide range of imaging agents and because of this do not use them in their research practice and do not initiate clinical trials. Finally, radiopharmaceutical companies whose funding is essential for crossing the “death valley” between the laboratory research and clinical investigation have not invested so far any significant funds and effort in bringing novel infection imaging agents into the clinic.

### Possible Ways Forward

Informative meetings of research and medical community with regulatory agencies such as the United States Food and Drug Administration (FDA) to provide the guidance on the preferred animal models of infections and the scope of preclinical work required for filing an Investigational New Drug (IND) application for a new infection imaging agent would greatly assist in moving novel infection imaging agents toward the clinic. In this regard, FDA and Nuclear Research Council (NRC) have recently conducted a series of joint webinars on alpha-emitter radio-therapeutic agents and the same format could be used for the infection imaging agents. There is also a need for the radiopharmaceutical scientists developing new infection imaging agents to establish connections with the infectious disease physicians and to inform them about the new agents and opportunities for collaborative research and clinical trials. Ongoing COVID-19 pandemic has demonstrated to the biotechnology and pharmaceutical industries that there are ample business opportunities in the infectious diseases arena including patients diagnostic imaging. In addition, total body PET/CT greatly assists in new drug development by providing an opportunity to study the biokinetics of the radiolabeled forms of the drugs in all infectious foci in the body at the same time. Thus, we hope that current resurgent interest toward imaging of infections will provide the final push for concerted effort toward clinical testing of multiple promising infection imaging agents.

## Author Contributions

ED and DR performed the literature search. ED analyzed the data and wrote the first draft of the manuscript. All authors contributed to the article and approved the submitted version.

## Funding

The research was partially funded by the Fedoruk Center for Nuclear Innovation. We also thank the National Council for Scientific and Technological Development (CNPq) of Brazil PQ1D302100/2018-0 to DR and to São Paulo Research Foundation (FAPESP) 2010/06374-1 to DR.

## Conflict of Interest

The authors declare that the research was conducted in the absence of any commercial or financial relationships that could be construed as a potential conflict of interest.

## Publisher's Note

All claims expressed in this article are solely those of the authors and do not necessarily represent those of their affiliated organizations, or those of the publisher, the editors and the reviewers. Any product that may be evaluated in this article, or claim that may be made by its manufacturer, is not guaranteed or endorsed by the publisher.
